# Endotoxic Septic Shock: Diagnosis and Treatment

**DOI:** 10.3390/ijms242216185

**Published:** 2023-11-10

**Authors:** Debra M. Foster, John A. Kellum

**Affiliations:** 1 Spectral Medical Inc., Toronto, ON M9C 1C2, Canada; kellum@pitt.edu; 2 Center for Critical Care Nephrology, University of Pittsburgh, Pittsburgh, PA 15213, USA

**Keywords:** endotoxin, sepsis, endotoxic septic shock, diagnosis of endotoxemia

## Abstract

Endotoxin, also referred to as lipopolysaccharide (LPS), is a potent stimulator of the inflammatory cascade which may progress to sepsis and septic shock. The term endotoxic septic shock has been used for patients who have a clinical phenotype that is characterized by high endotoxin activity in addition to a high burden of organ failure; especially a pattern of organ failure including hepatic dysfunction, acute kidney injury, and various forms of endothelial dysfunction. Endotoxic septic shock has been a target for drug therapy for decades with no success. A likely barrier to their success was the inability to quantify endotoxin in the bloodstream. The Endotoxin Activity Assay (EAA) is positioned to change this landscape. In addition, medical devices using adsorptive technology in an extra-corporeal circulation has been shown to remove large quantities of endotoxin from the bloodstream. Focusing on the use of EAA to determine high concentrations of endotoxin will allow patients with endotoxic septic shock to be identified quickly and these patients may benefit most from removal of endotoxin using extracorporeal methods.

## 1. Introduction

“*Endotoxins possess an intrinsic fascination that is nothing less than fabulous. They seem to have been endowed by nature with virtues and vices in the exact and glamorous proportions needed to render them irresistible to any investigator who comes to know them*”.

Dr. Ivan Loveridge Bennett Jr. 1964

Septic shock is a devastating disease with mortality that can exceed 50%. Even recent randomized trials of patients with septic shock in the US [1] and UK [2] found that mortality at 90 days was about 30%. Based on data using the Endotoxin Activity Assay (EAA) up to 80% of patients with septic shock have elevated levels of endotoxin, while at least 30% have very high levels, that is, >0.60 EA units [3] and experience mortality at roughly twice the rate of patients with septic shock without high levels of endotoxin [4]. One of the clinical challenges preventing the emergence of successful anti-endotoxin strategies has been to adequately identify patients with endotoxemia [5]. Elevated levels of endotoxin can occur in the setting of culture documented Gram-negative infection, from any source. It can also occur in the setting of Gram-positive infection, fungal infection, or in other cases of septic shock where no microbiologic source is identified as a result of translocation of endotoxin across the gut mucosal barrier in the setting of shock, hypoxemia, and gut hypoperfusion [3,6,7,8]. While it might seem logical to include patients with high suspicion of endotoxemia in clinical studies, lack of clinical diagnostic accuracy has contributed to the inability to prove overall efficacy of the treatment strategy proposed. The EAA is now being recognized as a necessary test for selecting appropriate patients that may respond to therapy [9]. This paper discusses the decades-long struggle to identify a treatment for endotoxic septic shock, a diagnosis associated with mortality > 50%, and an opportunity for new hope based on a new direction using EAA. 

## 2. Patient Identification for Anti-Endotoxin Therapy: Past and Future

Endotoxin, a lipopolysaccharide component of the cell wall of Gram-negative bacteria, has been a target therapy for sepsis for decades. However, despite promising preclinical data, past efforts targeting endotoxin as a treatment have failed, including human monoclonal IgM antibody against endotoxin, HA-1A [10], as well as its murine equivalent IgM, E5 antibody [11]. Furthermore, various endotoxin neutralizing proteins including bactericidal permeability increasing protein (BPI), analogues of Lipid A (E5564), and detoxifying agents such as alkaline phosphatase have failed [12,13,14]. Although intrinsic problems with these agents themselves may have contributed to their failure, no trial to date has tested an anti-endotoxin drug specifically in patients with proven endotoxemia. Studying a therapy in patients where only the minority can benefit requires large sample sizes and unrealistic effect sizes. For example, if only 30% of patients with septic shock have endotoxic septic shock, a trial of 2000 patients designed to detect a mortality difference would need to achieve at least a 24% absolute risk reduction for patients with endotoxic septic shock and have no adverse effect on mortality for the remaining patients. By contrast, a trial half this size would only require an 11% absolute risk reduction if it enrolled only patients with endotoxic septic shock. Failure to enroll the right patients may have obscured a survival benefit in prior trials. 

A current common theme in sepsis research is that better diagnostics are needed. This is because the complexity and heterogeneity of sepsis and the need to better target interventions to the right patient subset, at the right time, at an optimal dose and for an optimal duration, requires better diagnostics to first identify suitable patients to enable timely intervention where an intervention can impact outcome. Whereas the incidence of sepsis in the US is 1.5 million, with 1 in 5 developing septic shock [15], data from screening and enrolment into the Evaluating Use of Polymyxin B Hemoperfusion in a Randomized controlled trial of Adults Treated for Endotoxemia and Shock (EUPHRATES) trial [16] supports the segregated target sub-population with endotoxic septic shock as roughly half of this, or about 150,000 cases per year. 

Measuring endotoxin in whole blood or plasma is extremely difficult. Endotoxin is the major component of the cell wall of Gram-negative bacteria, comprising roughly 75% of the surface of the outer leaflet of the outer membrane of the cell wall. It is released into the circulation upon disruption of intact bacteria (death, cell lysis) infecting blood or tissues. It can also enter via translocating from the gastrointestinal tract when barrier function is compromised by hypoperfusion, inflammation, dysregulation of commensal flora, or sepsis from any source. Endotoxin is a glycolipid; it consists of a hydrophobic lipid part, called lipid A, which is anchored in the outer leaflet, and a hydrophilic polysaccharide part, which extends outside the cell. The polysaccharide part is composed of two domains: the core oligosaccharide and the O antigen. The O antigen (also called the O-chain) is a polysaccharide which is composed of several oligosaccharide units and is bound to lipid A through the core region. Lipid A deserves particular attention, as this part of the endotoxin molecule is sensed by the host and is responsible for activating the immune system [17]. 

Endotoxin that enters the bloodstream is rapidly sequestered by lipoproteins, mainly high-density lipoproteins (HDL) in cooperation with the phospholipid transfer protein (PLTP). Lipoproteins transport endotoxin to the liver, where it is inactivated by the enzymes acyloxy acyl hydrolase and alkaline phosphatase and excreted in the bile [18]. 

The most commonly used method for detecting endotoxin is the Limulus amebocyte lysate (LAL) assay. It is routinely used in the pharmaceutical industry to detect endotoxin contamination in crystalloid solutions and pharmacological preparations such as vaccines. However, LAL is not practical for use in whole blood and despite alterations to the LAL testing method to improve the test characteristics for blood, no LAL assay is available for clinical use [19,20]. There are numerous reasons that contribute to the difficulty in developing a blood test for endotoxemia. LPS binds to a number of plasma proteins, including lipopolysaccharide binding protein (LBP), high density lipoprotein (HDL), and to cellular blood components such as platelets. In addition, the molecular weight of endotoxin from different strains of Gram-negative bacteria can vary by 100-fold, making quantitative measures challenging. Furthermore, despite the availability of a detection IgM antibody, such as used in the EAA, development of an appropriate antibody immobilization strategy, such as may be used in an enzyme linked immunosorbent assay (ELISA) format, is a critical requirement in analytical performance, and to date has been elusive. 

Finally, there is heterogeneity among patients with sepsis not only with respect to endotoxin activity but also in the extent to which endotoxin produces disease [21,22]. Although humans are among the most sensitive species to endotoxin [23] they also possess a host of defenses [24]. In human volunteers, there is a characteristic hyper-inflammatory response such that upon endotoxin administration, there is a distinct, dose-dependent, and highly reproducible increase in plasma cytokine levels, tumor necrosis factor (TNF), and Interleukin-6 (IL-6) as well as the anti-inflammatory cytokine Interleukin-10 (IL-10). A major response is seen in the cardiovascular system with a decrease in mean arterial pressure and increased heart rate. While most patients with endotoxic septic shock are hyperdynamic with increased cardiac index, some patients also exhibit left ventricular dysfunction [25]. Removal of endotoxin therefore can have a dramatic effect on hemodynamics with improved mean arterial pressure and reduced requirement for vasopressors. 

In patients with septic shock, as endotoxin levels rise, immune defenses become overwhelmed but the threshold between survivable and lethal endotoxemia can be influenced by age and underlying comorbidity as well as genetic factors, particularly variation in genes regulating complement [26]. Manifestations of endotoxic septic shock such as coagulopathy and hyperlactatemia may help identify patients with lethal endotoxemia [27]. A sequential organ failure assessment (SOFA) score > 7 appears to delineate a high risk of death in patients considered for anti-endotoxin therapy [28,29]. 

## 3. The Endotoxin Activity Assay (EAA)

**Figure 1:**
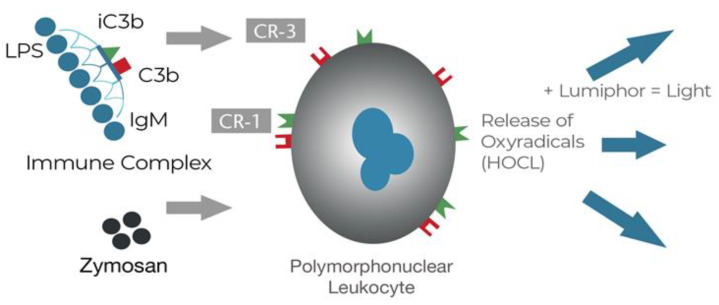
The EAA is a homogenous assay where the antigen-antibody immune complex reacts with the zymosan primed polymorphonuclear leukocyte to produce oxyradicals, that in the presence of a luminol, produce a photon of light. Reproduced from Romaschin et al. [9] *Crit. Care* **2012**, *16*, 248.

EAA is a homogeneous assay using an IgM antibody with a high affinity for the single epitope of Lipid A that then triggers the formation of an immunological complex including complement [30]. In a whole blood sample, endotoxin combines the IgM and the complex primes the complement receptors on the patient’s neutrophils to generate a release of oxyradicals. The test platform is chemiluminescence such that each oxyradical generated a photon of light produced by reacting with luminol, which were then counted in a luminometer (Figure 1).

**Figure 2:**
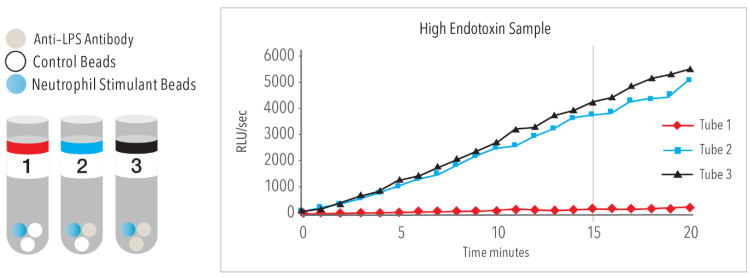
Whole blood from the patient is pipetted into 3 tubes and run in duplicate. Tube 1 is a “blank” and provides information on the baseline activation state of the patients polymorphonuclear leukocytes (PMN). In the example above, the Tube 1 (red line) has a very low reactivity. Tube 3 is a 1-point calibrator and contains an excess of Anti-LPS antibody. The graph above indicates the maximum reactivity for that patients PMNs. Tube 2 is the sample tube with a fixed concentration of anti-LPS antibody. In the graph above, the sample contains a large amount of LPS such that the Tube 2 reactivity is close to the maximum. This sample indicated a high level of endotoxin activity. The unitless results are expressed as from 0 to 1.0 based on the formula T2 − T1/T3 − T1.

The EAA is composed of 3 tubes, [Figure 2] where Tube 1 is a “blank” and provides information on the baseline chemiluminescence in the whole blood of the individual patient sample. Tube 3 is a 1-point maximum calibrator that represents maximal stimulation with an excess of antibody. Tube 2 is the “sample” containing a fixed concentration of antibody. The EAA result is expressed as a relative value between the sample minus the blank and the maximum calibrator minus the blank [Tube 2 − Tube 1/Tube 3 − Tube 1]. The EAA level is based on a scale of 0 to 1, where 0 = no light emission and therefore no endotoxin activity and 1 represents 100% maximum activity which is based on the concentration of antibody and a fixed concentration of *E. coli* 011B5. EAA results 0–4 indicates low endotoxin activity, 0.4–0.6 are intermediate levels, and EAA > 0.6 are high levels. 

**Figure 3:**
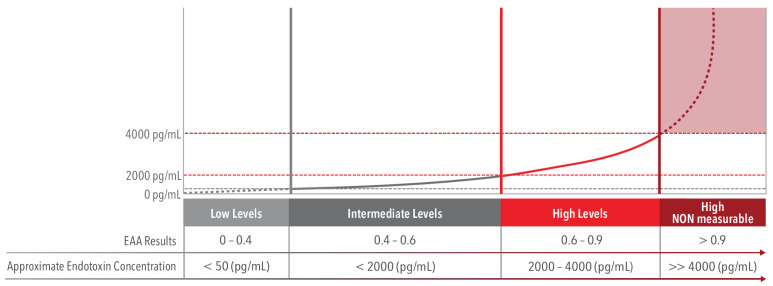
This representative dose response curve is logarithmic with a y-asymptote. Low EAA results (<0.04) are approximately equivalent to an LPS concentration of <50 pg/mL. Intermediate levels (0.4–0.6) are approximately equivalent to an LPS concentration of <2000 pg/mL. A high EAA level (0.6–0.9), is approximately equivalent to 2000–4000 pg/mL. For EAA results that are >0.9, it is beyond the limits of the assay to accurately determine a LPS concentration equivalent; however, it may be substantially higher than 4000 pg/mL. Adapted from Romaschin, A.D. et al. [31] *Blood Purif*. **2017**, *44*, 193–197.

Given the format of the assay, it is helpful to show the interpretation of the unit-less EAA against a representative dose response curve to approximate an EAA level with a concentration of endotoxin in picogram/mL. In Figure 3, an EAA result of 0.6 is approximately 2000 pg/mL and EAA 0.9 is approximately 4000 pg/mL. For EAA levels above 0.9 it is impossible to equate a measurable estimate >>4000 pg/mL which is a large, often lethal concentration of endotoxin [31]. Therefore, EAA levels in the range of 0.6 to 0.9 is the optimal interval when selecting a patient for treatment with the PMX cartridge. 

The clinical utility of the EAA was first established in the Multicenter endotoxin detection in critical illness (MEDIC) trial [3]. Marshall and colleagues performed a multi-center multi-national study of 856 patients upon admission to the intensive care unit (ICU). The results of this study showed that endotoxin activity was significantly correlated with risk of developing sepsis, or septic shock as well as mortality. More recently, Adamik and colleagues found that among patients with septic shock, those with high endotoxin activity experience mortality of approximately 60% while patients with septic shock but without high levels of endotoxin have only a 30% mortality [4]. Thus, using the EAA together with clinical assessment, a patient population with endotoxic septic shock can be identified as an appropriate population for an anti-endotoxin therapy. 

Furthermore, direct endotoxin removal strategies that have been tested clinically, such as the neutralizing protein BPI and analogues of Lipid A such as E5564, and others may resurface with the ability to properly select for patients with endotoxemia using the EAA. 

While endotoxin has both direct cytotoxic effects as well as effects mediated by the immune system (and complement) an approach of treatment directed at endotoxin may have greater effect in combination with the downstream effects on the immune response. To date, targeting cytokines TNF and IL-1 for example, have not shown a mortality benefit alone although they may have a role when added to treatments directed at endotoxin.

## 4. Removal of Endotoxin with Extracorporeal Blood Purification

While drug development continues, an alternative strategy aims to remove endotoxin directly from the blood [32,33,34,35,36]. The PMX cartridge (TORAYMYXIN PMX-20R [adult], and TORAYMYXIN PMX-05R [pediatric]) is an extracorporeal hemoperfusion cartridge intended for the selective removal of endotoxin from circulating blood through direct hemadsorption. The PMX cartridge consists of a housing fabricated of polypropylene packed with polystyrene fibers which is used as part of an extracorporeal hemoperfusion system. Polymyxin B is chemically immobilized on the polystyrene fiber with a density of 3.4 mg/g fibers. Each cartridge contains at least 190.4 mg of irreversibly bound Polymyxin B. This Polymyxin B adsorbs and removes endotoxin from the circulating blood as it passes through the cartridge. Each cartridge contains 56 ± 3 g fibers (dry weight) and has a blood volume capacity of 135 ± 5 mL. 

A pub-med search in January of 2023 for Polymyxin-B hemoperfusion showed that over 460 articles have been published in over 30 years since the first publication on PMX appeared in 1989 [37]. Since the first published report of PMX use clinically, there have been more than 270 articles reporting clinical and observational studies including case studies, of PMX use in approximately 27,300 critically ill patients, of whom more than 14,500 received at least 1 “dose” (cartridge) and up to 7 doses of the PMX cartridge via hemoperfusion. This represents approximately 29,000 episodes of exposure to the PMX cartridge in the published literature alone.

Aoki, et al., at Shiga University, Japan, performed first in animal studies using polymyxin-B adhered to polystyrene derivative fibers with a cartridge using hemadsorption [38]. Dogs were given a dose of 0.75 mg/kg endotoxin then hemoperfusion with PMX at 50 mL/min. The animals showed an immediate severe decrease in blood pressure, white blood cell (WBC), and platelet count following endotoxin administration. However, animals receiving PMX recovered 80% of the comparative initial blood pressure and WBC and platelet counts fully recovered immediately upon completion of hemadsorption. Survival to 7 days was 73% (11/15) for dogs in the PMX group compared with 10% (1/10) for controls [39].

## 5. Clinical Studies of PMX

**Figure 4:**
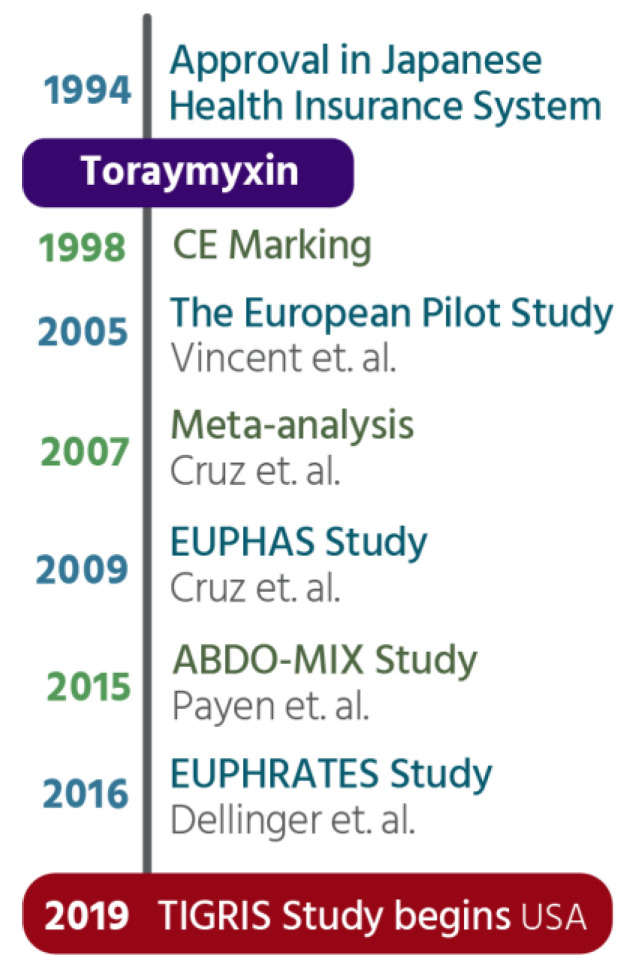
Provides a vertical timeline for clinical milestones associated with use of the PMX cartridge. It was approved for clinical use in Japan in 1994 under the trade name Toraymyxin. The device achieved CE marking in 1998 then underwent a series of clinical studies in Europe that were published in 2005 [41], 2009 [42] and 2015 [43] with a meta-analysis published in 2007 [40]. The PMX cartridge underwent a North American study, EUPHRATES, published in 2016 [16] and is currently undergoing a randomized clinical study, Tigris, in the US that began in 2019.

The effectiveness of endotoxin removal using the PMX cartridge has been shown in numerous studies. Cruz et al., published a systematic review of the literature on the effects of the PMX cartridge in patients with sepsis [40]. The meta-analysis included 28 publications between 1998 and 2006 with a pooled sample size of 1425 patients, 978 of whom received the PMX cartridge and 447 received standard medical care alone. Most studies were performed in Japan, then Western Europe prior to North America based studies [Figure 4]. The major finding was that PMX therapy was associated with significantly lower mortality risk (Relative Risk 0.53, 95% CI, 0.43–0.65, *p* < 0.001) based on the pooled data for hospital mortality (61.5% in the standard medical care group and 33.5% in the PMX cartridge group). This dramatic reduction in mortality was accompanied by an increase in mean arterial pressure of 19 mmHg (95% CI, 15–22 mmHg, *p* < 0.001) representing a 26% mean increase in MAP (range 14–42%). Concomitantly, there was a decrease in dopamine/dobutamine dose by 1.8 μg/kg per minute (95% CI, 0.4 to 3.3 μg/kg per minute; *p* = 0.01) after PMX use. The mean PO_2_/FiO_2_ ratio increased by 32 units (95% CI 23–41, *p* < 0.001). For patients who received PMX, endotoxin levels decreased by 21.2 pg/mL (95% CI, 17.5–24.9 pg/mL *p* < 0.001) compared with endotoxin levels in patients receiving standard medical care. This represents a decrease in endotoxin of between 33% and 80% from pre-PMX treatment levels. 

Vincent and colleagues conducted the first European multi-center randomized trial for PMX use in intraabdominal sepsis [41]. Subjects were randomized to receive either PMX hemadsorption (n = 17), or standard of care (n = 19). Use of the PMX cartridge was limited to a single cartridge only. While, the study was not powered for a mortality difference there were changes in cardiovascular status such that increased mean arterial pressure (6 mmHg ± 13.6 from baseline to day 2, *p* = 0.006), increases in cardiac index (CI; *p* = 0.012 and 0.032 at days 1 and 2, respectively), left ventricular stroke work index (LVSWI, *p* = 0.015 at day 2), and oxygen delivery index (DO2I, *p* = 0.007 at day 2) compared with the controls. The need for continuous renal replacement therapy (CRRT) after study entry was also reduced in the PMX group (*p* = 0.043). 

In 2009, Cruz et al. published a multi-center randomized trial in Italian centers comparing PMX to conventional medical therapy for 64 patients with septic shock from intra-abdominal infection [42]. The primary outcome was change in MAP and vasopressor requirement over 72 h, both of which were significantly impacted by PMX. Furthermore, the secondary outcome, 28-day mortality, was significantly reduced (32% PMX versus 53% conventional care, (unadjusted hazard ratio HR 0.42, 95% CI 0.20–0.94, adjusted for SOFA score, HR 0.36 95% CI 0.16–0.80, *p* = 0.012)). Furthermore, at 72 h post-treatment the patients treated with PMX had a significant improvement in a composite organ failure score (SOFA delta −3.4 [95% CI, −4.4 to −2.4] compared with patients receiving conventional therapy alone (−0.1 [95% CI, −1.7 to 1.5], *p* < 0.001)).

In 2015 Payne et al. reported on a similar trial in France [43]. This study, Early use of polymyxin B hemoperfusion in patients with septic shock due to peritonitis (ABDOMIX), was an open-label, unblinded, multicenter randomized trial of PMX versus standard of care for subjects with septic shock immediately following abdominal surgery for intestinal perforation. The trial detected no difference in mortality at 28 days in 243 randomized patients. However, although the study was powered for a placebo mortality of 37% the observed mortality was only 19.5%. Thus, the study was substantially underpowered to detect a difference in the primary outcome and based on more recent evidence, may have enrolled a population unlikely to benefit from endotoxin adsorption. Furthermore, 38% of patients randomized to the PMX group did not complete both therapy sessions. In the majority of cases, this was related to cartridge clotting. No endotoxin measurements were performed prior to patient enrollment. However, in a post-hoc analysis of data from a subset of patients in the ABDOMIX study treated with the PMX cartridge, plasma samples were used to measure endotoxin mass concentration using an high performance liquid chromatography (HPLC) method [44]. Compared with prior studies from the same group [45], endotoxin was quite low for most patients in the ABDOMIX trial. Furthermore, the average SOFA score was 8 in both groups suggesting that the trial did not enroll a suitable patient population. A stepwise multivariate analysis validated simplified acute physiology score (SAPS) II and endotoxin levels as independent predictors of mortality at day 28. Patients with endotoxic septic shock and high SAPS II had the highest mortality.

The Early Use of Polymyxin B Hemoperfusion in a Randomized study of Adults Treated for Endotoxic Septic shock (EUPHRATES) trial was reported in 2018 [16]. The EUPHRATES trial also examined the impact of PMX on mortality in patients with septic shock but unlike other trials included endotoxemia, defined as EAA ≥ 0.60, as an entry criterion. At interim analysis, the median Multiple Organ Dysfunction Score (MODS) was 9 (equivalent to a SOFA of 11) and no benefit from PMX was observed for patients in the lower half of the MODS distribution. Therefore, the inclusion criteria were changed to include only patients with MODS > 9. However, 28-day all-cause mortality was not significantly different for patients with EAA ≥ 0.60, MODS > 9 and receiving two doses of PMX compared with sham hemadsorption (38/115, 33% PMX vs. 47/129, 36.4%, absolute difference −3.1%, *p* = 0.58). 

For patients with EAA values ≥ 0.90, the endotoxin activity burden may be very high with up to 100-fold variability and is potentially unquantifiable; these patients are potentially less likely to respond to a standard regimen of two PMX treatments. When these patients were removed from the analysis, there became a mortality benefit for patients with MODS > 9 in the PMX arm at 28 days, 23 patients of 88 (26.1%) died versus 39 of 106 (36.8%) in the sham group (absolute difference 10.7%, OR 0.52, 95% CI (0.27, 0.99), *p* = 0.047) [46].

## 6. Adsorptive Capacity of the PMX Cartridge and the Tigris Trial

The results from the EUPHRATES trial added two new dimensions to patient selection for endotoxin removal. First, as seen with subsequent studies [28,29] severity of organ failure is a determinant of patient response. Second, the burden of endotoxin may be too high for some patients to benefit. This later aspect was explored by Romaschin and colleagues who tested the adsorption capacity of PMX-20R cartridges in a study using matrices and conditions relevant to those used in patients for therapeutic purposes [31]. During production of the PMX-20R cartridge a quality control measure test is performed to validate production lots prior to release. Using endotoxin-spiked Bovine plasma, the minimum release specification is 60% removal of a total load of 15 μg of endotoxin. Romaschin found that in 52 cartridges tested, there was 88% removal or an average of about 12 μg of *E. coli* O111:B4 endotoxin. Therefore a 2-cartridge dosing regimen would provide a total binding capacity in the range of 10–20 μg of endotoxin. However, an EAA > 0.9 equates to a concentration of endotoxin >4000 pg/mL or >20 μg assuming a 5 L blood volume. Thus, >0.9 represents the upper limit of detoxification capacity for two PMX cartridges even if the endotoxin load is stable. 

There are other adsorptive cartridges on the market that claim to remove endotoxin. However, the PMX cartridge has the highest binding capacity and therefore the most rapid and effective removal [32,47]. It is specifically designed to adsorb endotoxin for removal from the bloodstream. 

The above considerations led to the design of the Tigris trial (NCT 03901807). Tigris is an ongoing clinical trial using the PMX cartridge that is designed to confirm the subgroup analysis from the EUPHRATES trial. In the Tigris study, patients are selected based on an EAA level of 0.6–0.9 together with MODS > 9 or SOFA > 11. The trial will enroll 150 patients randomized 2:1 (100 PMX and 50 control). The Statistical analysis plan for this study includes a Bayesian approach, building on data collected in the EUPHRATES Trial. 

Future studies may elucidate the effect of a combined strategy of endotoxin targeted removal plus a non-specific cytokine removal therapy. 

## 7. Recent Observational Studies

Real world experience with the PMX cartridge can be evaluated using data from the EUPHAS2 Registry. The EUPHAS2 registry aims to collect data on the daily use of PMX in critically ill patients with septic shock, in order to identify specific patient populations that may benefit from this therapy and provide proof of concept for future trials. There are approximately 35 hospitals contributing more than 400 patients. A recent study from Registry patients consisted of those patients that are considered EUPHAS-like, meaning abdominal surgery patients treated with two cartridges and had EAA levels > 0.60 [48]. Interestingly the 28-day mortality in this real-world cohort was 36%, similar to that of the EUPHAS study treated group at 32%. There were also comparable positive effects on SOFA scores, and its organ-specific components cardiovascular and renal SOFA. 

A contemporary review from Japan of results on mortality and organ function effects was performed using data from the Japanese diagnosis procedure combination database from 2016 to 2018 [49]. In this analysis, 4766 received PMX and propensity score matching produced a matched cohort of 4141 pairs with well-balanced patient backgrounds. The 28-day mortality rate was 22.1% in the PMX group and 28.9% in the control group (*p* < 0.0001). Median noradrenalin-, dialysis, and ventilator-free days were 2 days (*p* < 0.0001), 2 days (*p* < 0.0001), and 6 days (*p* < 0.0001) longer in the PMX group than in the control group, respectively.

## 8. Conclusions

Nearly sixty years ago, Dr. Ivan Loveridge Bennett Jr., wrote, that “*It has been known since the last century that the injection of Gram-negative bacteria or their products will produce fever in man. Other striking alterations include polymorphonuclear leukopenia followed by leukocytosis, enhanced reactivity to epinephrine and related substances, hemodynamic changes, increased or decreased resistance to infection by bacteria and viruses, destruction of tissue as exemplified by the Shwartzman reaction, potentiation of antibody response, depletion of liver glycogen, shock and vascular collapse, and, in sufficiently large dosage, death*” [50].

Today there is renewed optimism for finding a successful treatment for endotoxic septic shock. By identifying patients with a high but treatable burden of endotoxin (EAA 0.6–0.9) together with a high burden of organ dysfunction we can select patients most likely to benefit from PMX and possibly other treatments. Use of these therapies in the real world will also be most effective in such patients but use in patients with EAA > 0.9 may be justifiable as may use in patients with lower EAA (e.g., 0.5) if producing profound organ dysfunction. The ultimate solution for patients with endotoxic septic shock remains to be rapid diagnosis and treatment. This paper illustrates one example of using the EAA in combination with the PMX cartridge. The future may provide greater opportunities to target endotoxin such as by pharmacologic or extracorporeal methods or combinations thereof, to further reduce mortality [32,51].

## Data Availability

This manuscript did not report any new data.
